# When Everyday Sounds Become Barriers: The Effect of Misophonia on Speech Perception

**DOI:** 10.1002/brb3.70924

**Published:** 2025-10-21

**Authors:** Nazife Öztürk Özdeş, Suna Tokgöz Yılmaz

**Affiliations:** ^1^ Audiology and Speech Disorders Department, Institute of Health Sciences Ankara University Ankara Turkey; ^2^ Audiology, Balance and Speech Disorders Diagnosis and Rehabilitation Unit, Ibn‐i Sina Hospital Ankara University Faculty of Medicine Ankara Turkey; ^3^ Audiology Department Faculty of Health Sciences Ankara University Ankara Turkey

**Keywords:** auditory processing, misophonia, speech perception in noise, triggering sounds

## Abstract

**Introduction:**

Misophonia is a condition characterized by intense emotional reactions, such as anger, anxiety, or disgust, in response to specific sounds. This study aims to investigate the speech perception performance in noise of individuals with misophonia. Recent perspectives suggest that these emotional reactions may interfere with auditory attention, particularly in socially relevant listening situations. However, little is known about how misophonia affects speech perception in noisy environments.

**Methods:**

The study included 40 individuals with misophonia and 40 healthy controls, matched for age and gender. Both groups were administered the Hearing in Noise Test (HINT) under two different scenarios: one with speech noise only and another with speech noise combined with the triggering sound of a buzzing fly. The fly sound was identified as aversive by all participants with misophonia. Speech perception performances in noise of the groups across the two scenarios were compared.

**Results:**

The findings revealed that the presence of a triggering sound significantly impaired the speech perception ability in noise in individuals with misophonia. The misophonia group demonstrated lower performance in the presence of the triggering sound compared to the control group. Additionally, increased severity of misophonia and a greater number of triggering sounds were associated with further declines in HINT performance.

**Conclusion:**

This study highlights that misophonia is a condition that adversely affects speech perception in noise. Understanding the communication challenges faced by individuals with misophonia in noisy environments provides a crucial foundation for the assessment of this disorder and the development of therapeutic interventions.

## Graphical abstract

Misophonia impairs speech perception in noisy environments, especially when triggering sounds are present. The severity of misophonia and the number of trigger sounds further worsen this effect, highlighting the disorder's impact on real‐life communication.

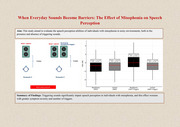

## 1 Introduction

Misophonia is a condition characterized by intense emotional reactions, such as anger, anxiety, or disgust, in response to specific sounds typically produced by others, rather than by the individuals themselves (Edelstein et al. 2013). The condition typically manifests during adolescence or early adulthood and seems to be reported more often among females (Wu et al. 2014; Jager et al. 2020). While the prevalence of misophonia differs among studies, recent research indicates that roughly 20% of the general population may encounter some type of misophonic symptoms, with clinical‐level misophonia estimated to be about 5% (Jager et al. 2020).

Recent perspectives highlight that the emotional effects of these triggering sounds can be greatly affected by the social context and the perceived acceptability of the actions that generate these sounds, rather than being determined exclusively by their acoustic characteristics (Berger et al. 2024; Norena 2024).

While the neurophysiological foundations of misophonia remain incompletely elucidated, research indicates that this condition may stem from disturbances in the neural integration of auditory processing and emotional regulation networks (Jastreboff and Jastreboff 2023). Neuroimaging studies reveal atypical activation patterns in brain areas such as the anterior insula and anterior cingulate cortex, which play essential roles in emotional processing, interoception, and salience detection (Kumar et al. 2021; Schröder et al. 2019). Additionally, Berger et al. (2024) suggest that misophonia should be understood within a more extensive framework of social cognition, emphasizing that emotional responses to sounds are influenced by context and shaped by social interpretation.

Norena also points out that misophonia may be driven by social conditioning and the perception of certain sounds as socially inappropriate or offensive, contributing significantly to emotional distress and social withdrawal (Norena 2024). Given this, misophonia could have implications for speech perception in noisy social environments, potentially interfering with cognitive resources required for effective communication. Specifically, the strong emotional responses associated with misophonia are likely to redirect attentional and cognitive resources away from tasks involving auditory processing. A research study indicated that people suffering from misophonia demonstrated a diminished capacity for selective attention when they were subjected to trigger sounds that they perceived as bothersome (Silva and Sanchez 2019).

Consequently, this increased emotional reactivity may adversely affect the capacity of individuals with misophonia to concentrate on speech signals in the presence of background noise, resulting in diminished performance in speech‐in‐noise (SiN) situations. However, few studies have systematically explored how misophonia affects auditory speech processing in realistic scenarios. A study conducted by Kim et al. (2023) assessed the SiN capabilities of individuals suffering from misophonia and hyperacusis. The results revealed that the misophonia cohort demonstrated inferior SiN performance when compared to both the control and hyperacusis groups at signal‐to‐noise ratios (SNRs) of 20 and 5 dB. Additionally, another study examining the audiological characteristics of adults with misophonia found that approximately 15% of participants had mild SNR loss (Muñoz et al. 2024). However, apart from these studies, there exists a notable lack of research examining the specific effects of misophonia on speech perception in noisy environments. This deficiency constrains our comprehension of how emotional and cognitive reactions to triggering sounds might disrupt auditory understanding in socially significant listening situations.

This study aims to evaluate the speech perception performance in noise of individuals with misophonia and investigate the effects of the number of misophonic triggering sounds and the severity of misophonia on this performance. The variability in individuals' responses to trigger sounds necessitates the consideration of both symptom severity and the number of triggering sounds. This evaluation seeks to contribute to a better understanding of the condition.

## 2 Materials and Methods

The participants included 40 adult individuals with misophonia, who presented with complaints of sound sensitivity at the Audiology, Balance, and Speech Disorders Unit of Ankara University Faculty of Medicine, Ibn‐i Sina Hospital, and 40 healthy controls matched for age and gender. The total sample consisted of 80 adults, with 40 individuals in the Misophonia Group (MG) and 40 individuals in the Control Group (CG). The participants ranged in age from 19 to 64 years (*M* = 42.75, SD = 12.18). The sample was predominantly female, with 90% women (*n* = 72) and 10% men (*n* = 8). For the MG, the mean Misophonia Questionnaire (MQ) score was *M* = 32.25 (SD = 14.35), and the mean number of misophonic triggers was *M* = 19.75 (SD = 14.44). A descriptive overview of misophonia scores is given in Table 1. All participants were native Turkish speakers, had normal hearing thresholds (< 16 dB HL from 125 to 16,000 Hz), and met the respective inclusion criteria for their group.

**TABLE 1:** Descriptive overview of misophonia scores.

Measure	Mean	SD	Min	Median	Max
MQ score	32.25	14.35	6.00	29.50	63.00
HQ	8.5	3.5	0.0	9.0	14.00
LDLmin	90	9.5	85	89	120
MSL score (Number of triggers)	19.75	14.44	1.00	18.00	50.00

Abbreviations: HQ, Hyperacusis Questionnaire; LDLmin, loudness discomfort level minimum value; MQ, Misophonia Questionnaire; MSL, Misophonia Symptom List.

Written and verbal informed consent was obtained from all participants. Ethical approval for the study was granted by the Ankara University Human Research Ethics Committee on October 10, 2024 (approval number: İ09‐659‐24). The research was conducted in accordance with the principles of the Helsinki Declaration.

All participants underwent routine audiological assessments and evaluations for decreased sound tolerance. To distinguish decreased sound tolerance, the Misophonia Symptom List (MSL) (Kılıç et al. 2021), MQ (Sakarya and Çakmak 2023), Khalfa Hyperacusis Questionnaire (HQ) (Erinc and Derinsu 2020), and loudness discomfort level (LDL) measurements were used together.

### 2.1 Sample Size Determination

The sample size was calculated using the G*Power 3.1.9.7 software. Based on a Kruskal–Wallis test to compare four measurements (two different Hearing in Noise Test (HINT), results from the MG and the CG), a minimum of 68 participants was required to achieve 80% power, with a 0.05 Type I error rate and a medium effect size of 0.3 (Muñoz et al. 2024). Accordingly, the study was conducted with a total of 80 participants.

### 2.2 Inclusion Criteria

#### 2.2.1 Misophonia Group

‐Age between 18 and 65 years
‐Hearing thresholds of < 16 dB at each frequency between 125 and 16,000 Hz
‐Type A tympanogram findings and the presence of bilateral acoustic reflexes
‐Diagnosed with misophonia

Diagnostic criteria for misophonia were determined according to those suggested by Kılıç et al. (2021), and individuals in this group were included accordingly:

- The presence of at least one sound listed in the MSL is considered clinically significant and bothersome
- A marked emotional and/or physiological response when exposed to the sound
- Symptoms significantly impacting daily life

#### 2.2.2 Control Group

‐Age between 18 and 65 years
‐Hearing thresholds of < 16 dB at each frequency between 125 and 16,000 Hz
‐Type A tympanogram findings and the presence of bilateral acoustic reflexes
‐No diagnosis of misophonia (Kılıç et al. 2021).

### 2.3 Exclusion Criteria

#### 2.3.1 For Both Groups

‐Diagnosis of any known neurological or psychological disorders
‐Diagnosis or suspicion of central auditory processing disorder
‐HQ scores > 22 and minimum LDL values < 77, indicating hyperacusis (Erinc and Derinsu 2020). A total of 12 participants were excluded due to hyperacusis, based on the criteria.
‐Participants meeting these exclusion criteria were excluded from the study.

### 2.4 Audiological Evaluation and Scales

*Audiological evaluations* were conducted in sound‐treated rooms with ambient noise levels below 35 dB A, using an Interacoustics AC40 clinical audiometer and a GSI Tympstar Pro device, in compliance with Industrial Acoustic Company standards. These assessments included pure tone audiometry, speech audiometry, and immittance measurements, with hearing status determined through the integration of these data. Air conduction (AC) hearing thresholds were assessed using insert earphones for frequencies ranging from 125 to 8000 Hz and circumaural high‐frequency earphones for frequencies between 10,000 and 16,000 Hz. Bone‐conduction (BC) thresholds were measured at 500–4000 Hz frequency bands with a bone vibrator. Pure tone averages (PTA) were calculated as the mean of thresholds at 500, 1000, 2000, and 4000 Hz. Hearing loss severity was classified according to the AC PTA based on Goodman's 1965 classification. All participants were reported to have normal hearing levels.

*The MSL* is a validated 50‐item checklist of misophonic triggers (for instance, chewing gum, clicking a pen, a fly buzzing, or the sound of high heels) used to diagnose misophonia and identify triggering sounds (Kılıç et al. 2021). The *MQ* was employed to assess symptom severity. MQ scores range from 0 to 68, with higher scores indicating more frequent misophonia symptoms and increased negative emotional and behavioral responses to triggers (Sakarya and Çakmak 2023). While the MQ was not employed for diagnostic grouping due to the absence of a defined threshold score and lack of trigger specificity, it served as a valuable tool for assessing the severity and impact of misophonia symptoms in the MG.

*The Khalfa HQ* was used to differentiate misophonia from hyperacusis. HQ scores range from 0 to 42, with scores above 22 indicating hyperacusis (Erinc and Derinsu 2020). *LDLs* were measured in dB HL for frequencies between 125 and 8000 Hz to determine the intensity levels causing discomfort. This measurement was used to distinguish individuals with hyperacusis from other participants.

### 2.5 Evaluation of Speech Perception in Noise

*The HINT* evaluates the ability to recognize and repeat sentences under various noise conditions, providing an objective assessment of individuals' ability to understand speech in noisy environments encountered in daily life. The results of the test are presented as SNR, measured in decibels (dB). In the HINT, speech perception performance is expressed in terms of the SNR in dB. The higher SNR values indicate worse speech perception performance. Therefore, an increase in the SNR score reflects a decline in speech intelligibility in noise. The test can be conducted in four conditions: quiet, noise from the front, noise from the right, and noise from the left using an external loudspeaker (Soli and Wong 2008).

**FIGURE 1:**
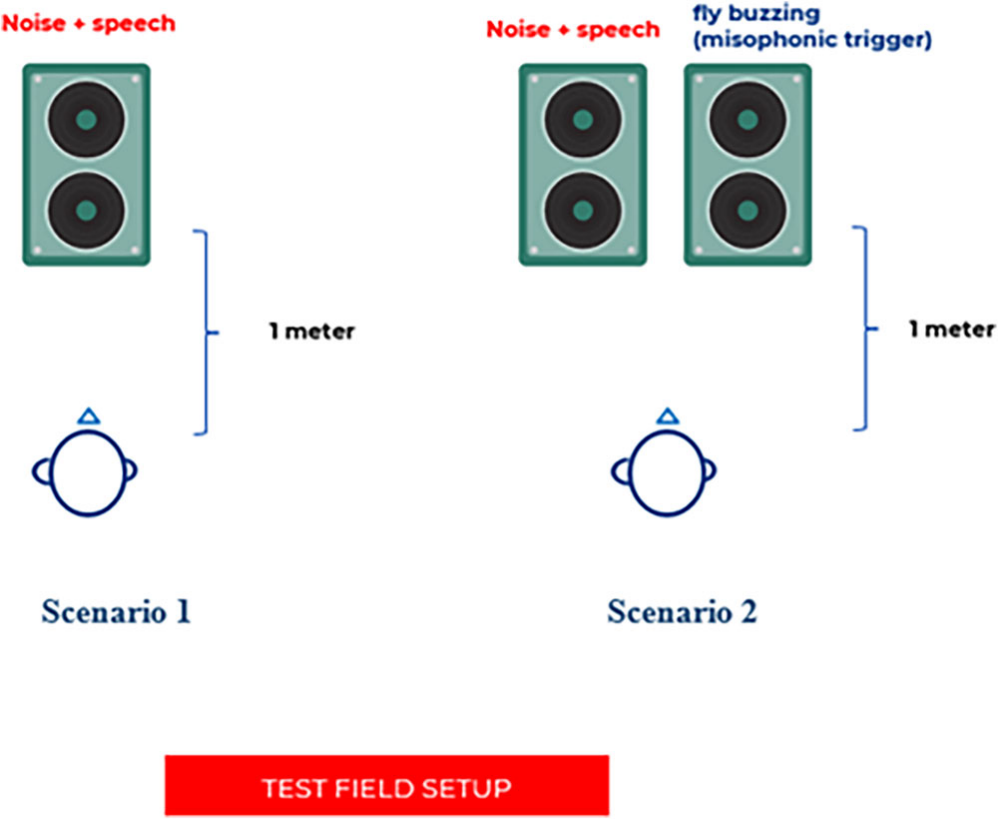
Implementation of the HINT procedure.

In this study, all participants completed the Turkish HINT (Cekic and Sennaroglu 2008) in an adaptive format under the noise front condition, applied in two different scenarios. In Scenario 1, the HINT test was administered with speech‐shaped noise (the test's default noise). In Scenario 2, the HINT test was conducted with speech‐shaped noise combined with a misophonic triggering stimulus (a buzzing fly sound). The background noise used in the HINT was speech‐shaped noise, which mimics the average frequency and intensity characteristics of human speech without containing actual words. It sounds like a steady, soft hissing or murmuring noise. The selection of the misophonic stimulus was based on the MSL, as the buzzing fly sound was identified as a common trigger among all MG participants. This choice aimed to best replicate the challenges individuals face in real‐life situations involving misophonic stimuli. The administration of the HINT test under both scenarios is illustrated in Figure 1.

All tests were conducted in a sound‐treated booth that complied with ANSI S3.1‐1999 standards, with background noise levels maintained below 35 dBA. Participants received the test via an external loudspeaker positioned 1 m in front of them at 0° azimuth.

The initial SNR was set at 0 dB and was adjusted in 2 dB steps based on the participant's correct or incorrect responses. Both the speech signal and the background noise (either speech‐shaped noise alone or speech‐shaped noise combined with a fly buzzing sound) were presented at 65 dB SPL.

Participants were asked to repeat each sentence aloud after hearing it. They were expected to respond immediately, with no fixed time limit but without undue delay. Only words that were repeated completely and accurately were scored. Each 20‐sentence HINT list took approximately 5 min to administer; thus, the duration per condition was around 15 min, and the total test time for both conditions was approximately 30 min. All test materials and procedures were based on the original study that developed the Turkish HINT (Cekic and Sennaroglu 2008).

### 2.6 Statistical Analysis

Descriptive statistics were presented using frequency, percentage, mean, and standard deviation. A Kruskal–Wallis *H* test was conducted to compare the hearing performance of the MG and CG across the two noise scenarios. Pairwise comparisons were performed using the Mann–Whitney *U* test and interpreted with Bonferroni correction. These nonparametric methods were selected due to the non‐normal distribution of the data. The Bonferroni correction was applied to adjust for the increased risk of Type I error associated with multiple comparisons. Relationships between numerical variables were assessed using Spearman's correlation test. A *p* value of < 0.05 was considered statistically significant.

## 3 Results

To compare the HINT performance of MG and CG participants in the presence and absence of the misophonic trigger (buzzing fly), the four HINT conditions (MG and CG under two noise scenarios) were analyzed using the Kruskal–Wallis test. The results indicated statistically significant differences in speech perception in noise performance between MG and CG (*H* = 13.718, df = 3, *p* = 0.003). To identify the source of these differences, pairwise comparisons were performed using the Mann–Whitney *U* test, with Bonferroni correction applied for interpretation.

### 3.1 Comparison of HINT Performance Between the Misophonia and CGs

The mean rank of HINT performance for MG was 38.65, compared to 42.35 for CG. In the standard HINT condition, the MG had a median SNR of −0.70 dB (IQR: −2.08 to −0.03), whereas the CG had a median SNR of −0.60 dB (IQR: −1.83 to 0.00). According to this finding, although individuals with misophonia had lower speech perception performance in noise than those in the CG, this difference was not significant. (*U* = 726.000, *Z* = −0.714, *p* = 0.475).

In the presence of triggering sounds, the mean rank of speech perception performance for MG was 48.60, while it was 32.40 for CG. This indicates that the speech perception performance of individuals with misophonia differed from the CG when triggering sounds were present, with MG exhibiting a higher mean rank. This difference was found to be statistically significant (*U* = 476.000, *Z* = −3.122, *p* = 0.002).

### 3.2 Within‐Group Comparisons

**FIGURE 2:**
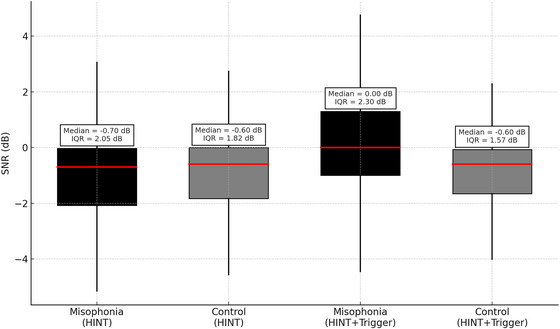
Comparison of HINT performance in the presence and absence of a misophonic trigger. “Boxplots represent the median signal‐to‐noise ratio (SNR) in dB for the Misophonia Group (black) and Control Group (gray) across two conditions: the standard HINT and HINT with a misophonic trigger (buzzing fly sound). Boxes indicate the interquartile range (25th–75th percentiles), horizontal lines within the boxes indicate the median, and whiskers represent the full range of non‐outlier values. Higher SNR values indicate poorer speech perception performance.”

The results indicated that the difference between the HINT and HINT with trigger measurements was statistically more significant in the MG than in the CG. In the HINT condition with the misophonic trigger (buzzing fly sound), the MG's performance decreased, with a median SNR of 0.00 dB (IQR: −1.00 to 1.30), while the CG maintained a comparable performance with a median SNR of −0.60 dB (IQR: −1.65 to −0.08). A significant difference was found between the HINT and HINT with trigger measurements in MG (*U* = 464.0, *p* = 0.0012), suggesting a notable distinction in the impact of the two test conditions on participants in this group. However, no statistically significant difference was observed between the HINT and HINT with trigger measurements in CG (*U* = 838.0, *p* = 0.718). Figure 2 illustrates the comparison of HINT performance between the groups in the presence and absence of the misophonic triggering sound.

Spearman's correlation analysis was conducted within the MG (*n* = 40) to examine the relationships between HINT performance and misophonia‐related features. The “number of misophonic triggers” refers to the total number of sounds marked as bothersome by each participant in the MSL. Misophonia severity was assessed using the MQ, with higher scores indicating greater symptom severity.

**FIGURE 3:**
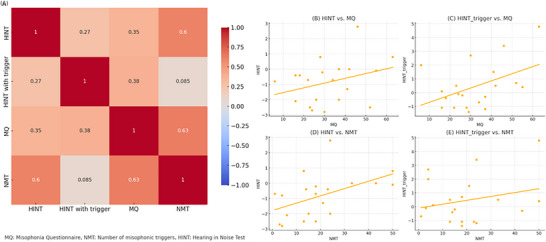
Associations between HINT performance, age, and misophonia‐related features. Spearman's correlation coefficients (*r* values) are presented in the matrix. The figure illustrates the relationships between standard HINT scores, HINT with misophonic trigger scores, misophonia severity (measured by the Misophonia Questionnaire), the number of misophonic triggering sounds, and age. Stronger positive correlations are indicated by darker shades. Statistically significant correlations (*p* < 0.05 or *p* < 0.01) suggest that higher misophonia severity and a greater number of triggers are associated with poorer speech perception performance, particularly under conditions involving misophonic stimuli.

A weak positive correlation was observed between HINT and HINT with trigger conditions (*r* = 0.269, *p* < 0.05). A moderate positive correlation was found between HINT scores and MQ scores (*r* = 0.352, *p* < 0.05), and a strong positive correlation was found between HINT scores and the number of misophonic triggers (*r* = 0.604, *p* < 0.01). Similarly, HINT with trigger scores showed a moderate correlation with MQ (*r* = 0.375, *p* < 0.05). A strong positive correlation was also identified between MQ scores and the number of misophonic triggers (*r* = 0.631, *p* < 0.01). These relationships are illustrated in the correlation matrix presented in Figure 3.

## 4 Discussion

This study aimed to evaluate the speech perception in noise performance of individuals with misophonia and to investigate the effects of the number of misophonic triggering sounds and the severity of misophonia on this performance. The findings provided significant insights into the cognitive processes and auditory perception associated with misophonia. While prior research had primarily focused on the emotional and behavioral consequences of misophonia, this study contributed to the limited body of literature exploring its neurophysiological effects and implications for auditory performance. In this context, the results were compared with existing literature, and the possible mechanisms underlying the findings were discussed.

Cortical auditory potentials, such as N1 and P1, are types of event‐related potentials (ERPs) that reflect time‐locked brain responses to auditory stimuli and are widely used to assess cortical‐level auditory processing. While some imaging and electrophysiological studies suggest no significant differences in early auditory processing in individuals with misophonia, other findings point to subtle alterations that may reflect higher‐order perceptual or emotional processing mechanisms. Among the limited studies investigating cortical auditory potentials in misophonia patients, one study (Schröder et al. 2014) reported a reduction in the N1 component, while another (Aryal and Prabhu 2024) demonstrated significant differences in the latency of P1 and N1 peaks. These differences suggest that the heightened sensitivity of individuals with misophonia to certain sounds may be related to disruptions at sensory gating levels within the auditory system. These findings indicate that misophonia may not be a direct auditory processing disorder but rather a condition associated with excessive emotional responses to auditory stimuli. This perspective aligns with the growing understanding of misophonia as a complex interplay between sensory processing and emotional reactivity (Aryal and Prabhu 2023).

There are limited studies on the speech perception in noise abilities of individuals with misophonia. In one study examining the audiological profiles of 60 adults with misophonia, the majority (97%) had normal hearing thresholds. However, 75% of participants reported self‐perceived hearing difficulties, and 15% demonstrated mild speech perception in noise difficulties as measured by the QuickSIN test (Muñoz et al. 2024). In another study involving an online assessment of individuals with misophonia, 22% reported challenges with hearing in noise (Enzler et al. 2021). Additionally, a study found that individuals with misophonia exhibited poorer SiN performance compared to those with hyperacusis and CGs (Kim et al. 2023). In our study, when speech perception in noise was evaluated with the combination of a triggering stimulus (buzzing fly sound) and speech noise, misophonic individuals performed significantly worse than controls, consistent with the literature. This finding highlights the negative impact of misophonia on speech perception in noise. However, when we evaluated with the standard HINT test, it was seen that the speech perception performance of misophonic individuals was similar to the controls. The comparable performance between misophonic participants and controls in the standard HINT test may be due to the inability of the test's speech noise stimulus to adequately represent the challenges experienced by individuals with misophonia in daily life. This suggests that the standard HINT may not fully capture the real‐life difficulties faced by individuals with misophonia.

In our study, to realistically assess the impact of misophonia on speech perception in noise, we applied HINT under two scenarios. In the first scenario, speech‐shaped noise was used, while in the second scenario, a buzzing fly sound reported as a triggering stimulus by all misophonic participants was added to the speech‐shaped noise. The findings demonstrated that speech perception in noise among individuals with misophonia is influenced by misophonic triggers. The significant differences between HINT and HINT with trigger measurements in misophonic participants suggest that these individuals may be more sensitive to misophonic triggers, which negatively impact their speech perception performance. This aligns with previous findings, where misophonic individuals performed worse than controls on a dichotic sentence identification test conducted with a misophonic auditory stimulus. The researchers highlighted that misophonic individuals might experience selective attention deficits when exposed to triggering sounds (Silva and Sanchez 2019).

Another study highlighted that speech perception in noise performance is largely determined by factors such as an individual's information processing capacity and short‐term memory capacity, provided their attention is not disrupted. It also emphasized that noise sensitivity could influence an individual's auditory attention management (Oberfeld et al. 2024; Lad et al. 2020). In our study, the decline in participant performance during the HINT with the misophonic auditory stimulus may similarly be related to selective attention and the utilization of cognitive resources. This suggests that misophonic triggers could interfere with the ability to effectively allocate cognitive and attentional resources in noisy environments.

The absence of a significant difference between the two measurements in the CG suggests that misophonic triggers do not have a notable impact on speech perception in individuals without misophonia. A previous study found a weak relationship between the degree to which short‐term memory is affected by irrelevant sounds and speech perception performance in noise (Oberfeld et al. 2024). The lack of influence from the triggering sound in the CG indicates that the cognitive processes involved in speech perception may differ between individuals with and without misophonia. This further supports the notion that misophonia may involve unique mechanisms affecting the allocation of cognitive resources in the presence of triggering stimuli.

Our correlation analyses revealed that as the severity of misophonia and the number of misophonic triggering sounds increased, HINT performance deteriorated. This finding suggests that the severity of misophonia symptoms may impact an individual's speech perception in noise, potentially leading to communication difficulties in daily life. The results align with the literature, indicating that individuals with misophonia may experience challenges in auditory attention and perceptual processes.

### 4.1 Study Limitations

Our study has several limitations. First, the sample size of 40 individuals with misophonia and 40 controls may limit the generalizability of the findings; a larger and more diverse sample could provide more robust conclusions. Additionally, the HINT test utilized only one misophonic trigger. Future studies employing a variety of triggering sounds could offer a more comprehensive evaluation of the effects of misophonia on speech perception.

Although the MSL is validated and culturally adapted, its diagnostic criterion—classifying misophonia if at least one sound is rated as moderately or severely disturbing—may include individuals with subclinical sound sensitivity and reduce specificity for clinically significant misophonia. Furthermore, while the use of a single common trigger (fly buzzing) ensured consistency across participants, it may not fully reflect the diversity of clinically prominent triggers, such as orofacial eating or breathing sounds, which are more representative of daily‐life misophonia experiences. These factors should be considered when interpreting and generalizing our findings.

The cross‐sectional design of our study does not allow for the observation of changes in misophonic responses or adaptation processes over time. Longitudinal studies could address this limitation. Moreover, incorporating physiological response measurements (e.g., heart rate, skin conductance) in future research could enable a deeper investigation into the physiological underpinnings of misophonic reactions.

## 5 Conclusion

This study represents an initial step toward evaluating the speech perception in noise performance of individuals with misophonia. The findings highlight that the presence of triggering sounds negatively impacts speech perception in noise among individuals with misophonia. Furthermore, the results demonstrate that the number of misophonic triggering sounds and the severity of misophonia exacerbate these negative effects on speech perception. These findings support the view that misophonia is not merely an emotional or psychological condition but also one that influences neurophysiological functioning. Additionally, this study provides valuable insights into the communication difficulties experienced by individuals with misophonia in noisy environments, offering important implications for the development of therapeutic interventions in the future.

## Author Contributions

Concept and design: Nazife Öztürk Özdeş, Suna Tokgöz Yılmaz. Literature search: Nazife Öztürk Özdeş. Data acquisition, data analysis, and statistical analysis: Nazife Öztürk Özdeş. Manuscript preparation: Nazife Öztürk Özdeş, Suna Tokgöz Yılmaz. Manuscript editing and manuscript review: Suna Tokgöz Yılmaz.

## Disclosure

The preliminary findings of this study were presented as an oral presentation at the seventh International Conference on Hyperacusis and Misophonia (ICHM7) on September 15–17, 2024.

## Ethics Statement

Ethical approval for the study was granted by the Ankara University Human Research Ethics Committee on October 10, 2024 (approval number: İ09‐659‐24). Additionally, individuals whose consent was obtained through the “Informed Volunteer Consent Form” were included in the study.

## Conflicts of Interest

The authors declare no conflicts of interest.

## Peer Review

The peer review history for this article is available at https://publons.com/publon/10.1002/brb3.70924

## Data Availability

Study data can be accessed upon request from the authors.
